# Ethylene Adsorption Using Cobalt Oxide-Loaded Polymer-Derived Nanoporous Carbon and Its Application to Extend Shelf Life of Fruit

**DOI:** 10.3390/molecules24081507

**Published:** 2019-04-17

**Authors:** Imam Prasetyo, Nur Indah Fajar Mukti, Teguh Ariyanto

**Affiliations:** 1 Department of Chemical Engineering, Universitas Gadjah Mada, 55281 Yogyakarta, Indonesia; 2 Advanced Material and Sustainable Mineral Processing Research Group, Universitas Gadjah Mada, 55281 Yogyakarta, Indonesia; 3 Department of Chemical Engineering, Islamic University of Indonesia, 55584 Yogyakarta, Indonesia; 165210101@uii.ac.id

**Keywords:** ethylene removal, porous carbon, storage life, synthetic polymer

## Abstract

Suppressing the amount of ethylene during storage has been of interest as a method to enhance shelf life of fruit. In this work, ethylene removal by adsorption using cobalt oxide-impregnated nanoporous carbon has been studied. Nanoporous carbon with a high surface area up to 2400 m^2^ g^−1^ was prepared by carbonization process biomass and synthetic polymer at 850 °C. Dispersion of cobalt oxide on porous carbon surface was carried out by an incipient wetness procedure followed by calcination process at 200 °C. Ethylene adsorption test was performed using a volumetric method in an ultrahigh vacuum rig constructed by Swagelok VCR® fittings. The results showed that the cobalt oxide/carbon system had significant ethylene adsorption capacity. Ethylene uptake increases with the increasing cobalt oxide loading on the carbon. The highest ethylene capacity of 16 mol kg^−1^ adsorbent was obtained by using 30 wt.% (weight percentage) of cobalt oxide dispersed in polymer-derived carbon. In closed storage, the ratio of 15 g adsorbent/kg fruit may extend the storage life up to 12 d, higher than that without adsorbent (3 d). Therefore, the results demonstrate the great potential use of cobalt oxide-impregnated nanoporous carbon as an adsorbent for ethylene removal during storage of fruit.

## 1. Introduction

Horticulture products like fruit typically have a short shelf life which limits their consumption, as well as lowers the economic returns of production. Therefore, several processes have attempted to improve postharvest storage while maintaining their quality. These include temperature management procedures [1], humidity management [2], and ethylene concentration control in the storage system atmosphere [3]. Ethylene is naturally generated by climacteric fruit during the process of ripening. The problem related to ethylene is that this hormone plant can promote the ripening process faster while at the same time it may also deteriorate the fruit. Therefore, for effective long-distance transport and subsequent storage, many fruit are required to be maintained at a low ethylene concentration environment. Due to its undesirable effect on the postharvest fruit, there has been large interest in removing ethylene from the storage environment to suppress its detrimental effect. 

Most commercial ethylene control systems are usually conducted by providing adequate ventilation, oxidizing ethylene with potassium permanganate, and removing ethylene with adsorbent materials [3]. Each of these methods has advantages and disadvantages or limitations. Maintaining ethylene concentration by using ventilation, for example, is not appropriate for a sealed environment. Hence, a number of efforts to remove ethylene from the packaging and storage atmosphere, by employing potassium permanganate particles as an oxidizing agent, have been presented in the literature [4,5,6]. However, ethylene oxidation using potassium permanganate will lead to the conversion of potassium permanganate to manganese oxide. Consequently, this ethylene oxidizing agent cannot be regenerated and reused. Furthermore, due to toxicity, the direct contact between permanganate and fruit is not allowed. Adsorption is another alternative method. It is conducted through introducing active material like palladium [7], cesium [8], and cobalt [9,10] on porous supports to scavenge ethylene. The latter approach is likely influenced by the type of active substance, material loading, and property of support material (e.g., specific surface area, pore size and pore size distribution) [11]. Among the active sites, cobalt oxide is attracted due to its strong adsorption affinity to organic substances [12,13,14]. Furthermore, it is relatively cheap compared to palladium. Our first attempt displayed the possible use of cobalt oxide for ethylene removal [9,15]. We believe that the support has strong affect, which is not yet explored for cobalt oxide in the application of ethylene removal. For instance, a low specific surface area of porous support hampers dispersion of the high content of active material which causes low uptake capacity. Hence, to date, searching for porous support for ethylene scavenger purposes is still challenging.

The paper presents a study of synthesis cobalt oxide dispersed on high-quality polymer-derived nanoporous carbon as an alternative method for controlling ethylene concentration in the atmosphere of packaging and storage systems. Porous carbon was selected due to its superiority in high content loading purposes compared to porous alumina or clay [16], and its lower affinity toward water than zeolite [17]. The preparation of material supports is the first step in the process development, which is then followed by characterization of the adsorbent in terms of its surface and ethylene adsorption properties. The effects of different carbon supports were also investigated by employing carbon synthesized from biomass. The capacity of ethylene adsorption and the efficacy of material in extending fruit storage life were studied by measuring ethylene adsorption and the storage life of fruit.

## 2. Results and Discussion

### 2.1. Adsorbent Characterization

**Figure 1:**
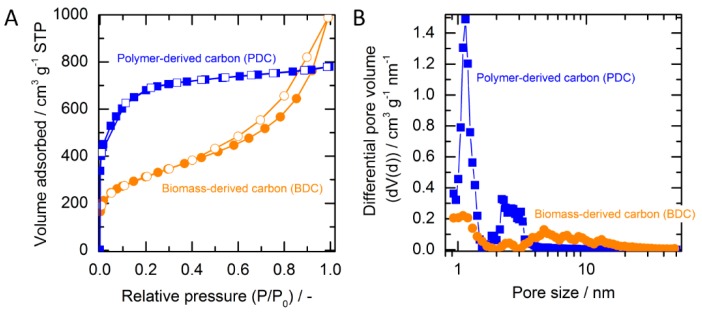
(**A**) N_2_-sorption isotherm (closed symbol: adsorption, open symbol: desorption). (**B**) Pore size distribution of carbon produced from biomass and synthetic carbon precursors.

Figure 1A displays the sorption isotherms of porous carbons obtained from the low-temperature nitrogen physisorption analysis. This is a standard procedure to characterize the porosity of a material. The adsorption and desorption branches are exhibited by closed and open symbols, respectively. The characteristics of the isotherm curve describes the type of porosity as in accordance with the International Union of Pure and Applied Chemistry (IUPAC) classification [18]. Biomass-derived carbon (BDC) exhibits a Type IV isotherm for mesoporous material showing an unrestricted monolayer-multilayer adsorption with a hysteresis loop. For polymer-derived carbon (PDC), a characteristic of adsorption for microporous material (Type I) is shown. This can be seen from the adsorption line which approaches a saturation value at a low relative pressure (P/P_o_). From the N_2_-sorption isotherm, it is clear that the produced carbons are porous. The pore size distributions based on the quenched solid density functional theory (QSDFT) model are shown in Figure 1B. BDC and PDC display remarkably different characters of pores. BDC possesses a broad pore size distribution in the range of micro- (<2 nm) and mesopore (2–50 nm) regimes. On the other hand, PDC displays a remarkable narrow and high signal (peak at 1.2 nm) and shows a portion pore in the range 2–5 nm. 

Structural properties (specific surface area, pore volume, microporosity, and average pore size) calculated from isotherm data are summarized in Table 1. For comparison, commercial activated carbon based on a coconut shell was also characterized and the data are also shown. Relatively, BDC and PDC exhibit better qualities of porous carbon materials when compared to the commercial carbon, and other porous carbons based on natural carbonaceous matters with a typical specific surface area of ca. 1000 m^2^ g^−1^ [19,20]. When comparing the characters of PDC and BDC, even the total pore volume is lower (1.21 cm^3^ g^−1^), and PDC (2390 m^2^ g^−1^) possesses a higher specific surface area than BDC (1080 m^2^ g^−1^). This is due to the substantial portion of micropores (60% by volume) and high pore volume of PDC. In Table 1, when cobalt oxide is deposited in PDC, the surface area drops to 1469 (15% Co/PDC) and 928 m^2^ g^−1^ (30% Co/PDC). The reduction is likely due to the weight of cobalt-oxide in the material, which is non-porous and possibly pore blocking by oxide particles. To further compare the porosity, an average pore diameter is given in Table 1. PDC and BDC exhibit ca. 2 and 5.7 nm mean pore diameter, respectively. Therefore, the property of the specific surface area exhibited by PDC is superior to that of the commercial carbon and BDC. It is worth mentioning that the PDC could compete with porous carbons synthesized from other polymeric materials which are typically in the range of 1000–2000 m^2^ g^−1^ [21] or novel porous carbon materials like carbide-derived carbons [22]. The porosity analysis also showed that the porous carbon obtained in the present study possessed good quality of carbon by having a specific surface area >1000 m^2^ g^−1^ which is the standard quality for carbon to be traded as commercial activated carbon. The high surface area and mesopores are important characters for successful impregnation of metal oxide on support.

**Figure 2:**
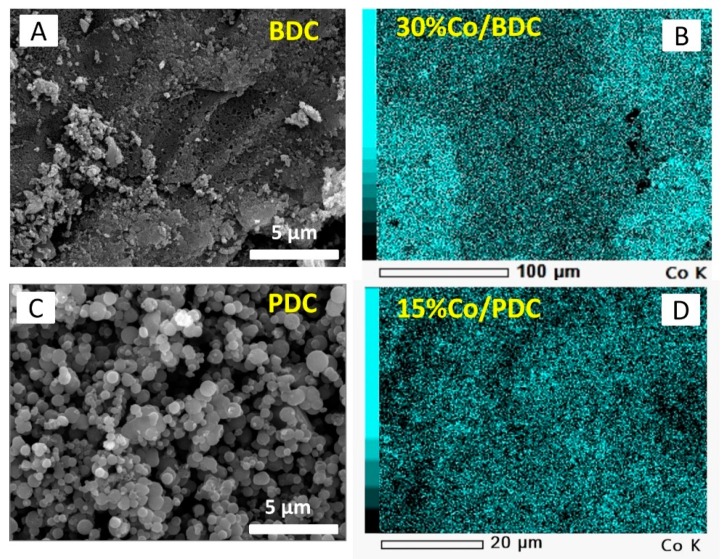
Scanning electron microscopy (SEM) micrographs of carbon supports (**A**,**C**) and their respective cobalt oxide-impregnated carbon materials (**B**,**D**).

The surface morphologies of the carbons obtained from pyrolysis of a mangosteen shell and phenolic resin were observed using scanning electron microscopy (SEM) and presented in Figure 2A,C, respectively. It can be seen that different carbon precursors produce a dissimilar surface morphology. BDC displays a rigid and dense structure which is typical of charcoal characteristics [23]. Whereas the PDC presents carbon spheres with a particle network producing a large void between spheres. BDC and PDC were then used as a carbon support of cobalt oxide. To observe dispersion of metal oxide on the carbon support, elemental mapping of SEM combined with Energy-dispersive X-ray (EDX) was carried out. Figure 2B,D show representatives of cobalt oxide loaded in BDC and PDC materials. All show a homogeneous dispersion of cobalt material (shown by cyan color) in porous carbon support. Furthermore, Co contents obtained by EDX analysis for 30% Co/BDC and 15% Co/PDC are 30.5 ± 1.2 and 14.7 ± 0.9 wt.%. It indicates that the target loading of element cobalt has been well achieved by a simple stoichiometric calculation as described in the impregnation methodology. In other words, the results above clearly show successful preparation steps to produce cobalt oxide porous carbon material.

### 2.2. Ethylene Adsorption

**Figure 3:**
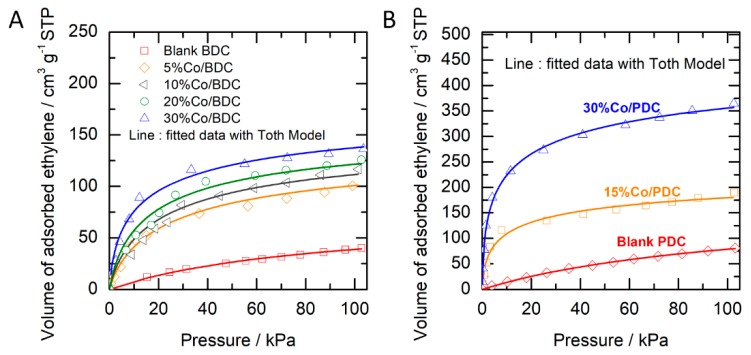
Adsorption isotherm of ethylene on cobalt oxide-impregnated porous carbon (data fitted with the Toth model). Case of carbon support: biomass-derived carbon (**A**) and polymer-derived carbon (**B**). Case (A) data taken from [10].

The performance of cobalt oxide-impregnated carbon material was tested for ethylene adsorption. Figure 3A shows ethylene sorption isotherm in BDC material with and without cobalt oxide in the range of 0–101 kPa pressure. The volume of adsorbed ethylene is defined as the volume at standard temperature and pressure (STP at 0 °C and 1 atm). In general, it can be seen that the ethylene uptake increases with increasing pressure. A significant amount of ethylene is adsorbed up to 40 kPa pressure. When increasing the pressure above 40 kPa, a minor amount of ethylene (an addition of 10%–25%) can be further adsorbed by all samples. For blank BDC, the uptake capacity is ca. 40 cm^3^ g^−1^ at 101 kPa. The introduction of cobalt oxide with 5% Co loading remarkably increases the ethylene adsorption, which results in ca. two times higher than the blank BDC. It clearly indicates that cobalt oxide material is very effective in enhancing ethylene adsorption. Increasing the content of cobalt oxide further increases the adsorption capacity of ethylene on cobalt oxide-impregnated porous carbon. The highest adsorption capacity of 135 cm^3^ g^−1^ or equivalent to 6.1 mol kg^−1^ is obtained when using 30%Co/BDC.

The performance of PDC with a remarkably high specific surface area as support material was then tested. The isotherm of ethylene adsorption in cobalt oxide/PDC is shown in Figure 3B. For blank PDC, the uptake capacity of 79 cm^3^ g^−1^ at 101 kPa was obtained. This is almost a double value when compared to blank BDC. The high specific surface area of PDC (two times higher surface area compared to BDC) likely corresponds to this remarkably adsorptive capacity of blank carbon. The result is in agreement with other studies that the higher the specific surface area, the higher the adsorption capacity of gaseous matters [20,24,25]. When introducing Co onto PDC, a remarkable enhancement of ethylene adsorption is achieved. Material with 15% Co and 30% Co content on PDC possess 180 and 350 cm^3^ g^−1^ at 101 kPa. The results show a boosting uptake of ethylene when using PDC as support. The high performance of Co/PDC might arise from the high specific surface area of the carbon support, hence a better dispersion of cobalt oxide particles may be achieved [26]. High dispersion leads to small particle sizes of active material, thus increasing adsorption sites [27]. When compared to another active site of cesium [8], cobalt oxide/BDC displayed a more or less similar performance. But, cobalt oxide/PDC exhibited a better system providing remarkably higher uptake capacity.

To qualitatively evaluate the adsorption performance, the isotherm data in Figure 3A,B were fitted by the Toth equation (Equation (1)) [28].

(1) $$C_{\mu }=C_{\mu s}\frac{bP}{{\left(1+{\left(bP\right)}^{t}\right)}^{\frac{1}{t}}}$$

The parameter of *C_μs_* is the maximum adsorption capacity while the parameters of *b* and *t* are the adsorption affinity and adsorption system heterogeneity, respectively. The optimal parameters of *C_μs_*, *b*, and *t* for the ethylene adsorption are presented in Table 2. It is clear that the higher the content of cobalt oxide, the higher the maximum adsorption capacity. Adsorbent of cobalt oxide dispersed on PDC displays a superiority of maximum uptake capacity (up to ca. 550 cm^3^ g^−1^). It appears that the adsorption of ethylene is more favorable in PDC material as indicated by a high value of the adsorption affinity (*b* value). This can also be qualitatively seen from the adsorption data (a remarkable adsorption of ethylene on PDC material at 10 kPa compared to on BDC at 30 kPa). When evaluating the *t* parameter, it seems that the system heterogeneity increases with introducing cobalt material in porous carbon since the value of *t* deviates further away from unity.

Adsorption temperature was varied from 293 to 313 K to evaluate sorption mechanism (see Supporting Information, Figure S1). The results showed that ethylene removal was more favorable at higher temperature suggesting chemisorption interaction between ethylene and cobalt oxide. This was then supported by the value of enthalpy of sorption of 74.1 kJ/mol which was in the range of chemisorption [29]. To show whether the adsorbent can be reused or not, repeatability experiments were performed. The results shown in Figure S2 of Supporting Information show that the adsorbent can be reused, but there is a slight reduction of uptake capacity. The adsorbent could maintain its capacity at 88% (Cycle 2) and 74% (Cycle 3).

### 2.3. Fruit Preservation Test

**Figure 4:**
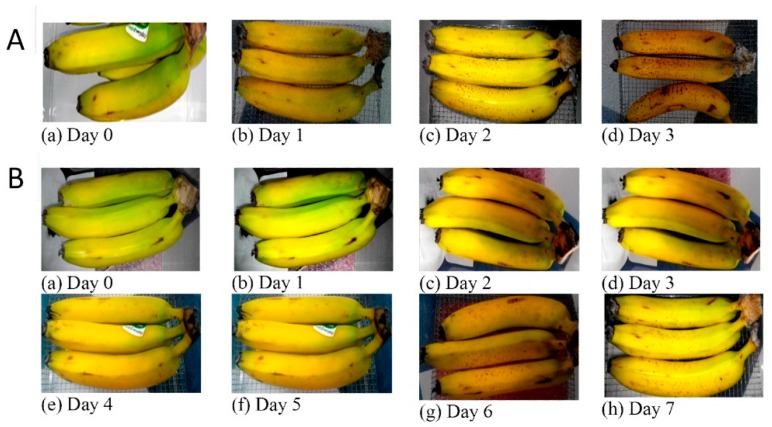
Images of Cavendish banana as the fruit model during the preservation test. Case of without adsorbent (**A**) and in the presence of adsorbent (**B**). Condition: 25 °C temperature and 80% relative humidity.

Figure 4A,B show day to day records of fruit conditions during a closed storage without and with cobalt oxide-impregnated carbon material (carbon adsorbent, 15%Co/PDC), respectively. The 15%Co/PDC material was used as it provides a high ethylene adsorption capacity of 175 cm^3^ g^−1^ with a mild content of cobalt oxide. The semiquantitative inspection was performed (i.e., color and brown area). The maximum preservation time was defined when brown color appeared dominantly, and bananas fell out from the bunch. When storing bananas in a closed box without carbon adsorbent, the fruit became rotten very quickly. Only on the third day, a single banana already fell out from the bunch and brown color emerged (see Figure 4A). With the carbon adsorbent, an extension of fruit preservation time can be clearly seen. The change in color was delayed with respect to the without adsorbent (Figure 4B). The results indicate the efficacy of cobalt oxide-impregnated carbon as an ethylene scavenger in a storage system, hence prolonging storage life of fruit.

**Figure 5:**
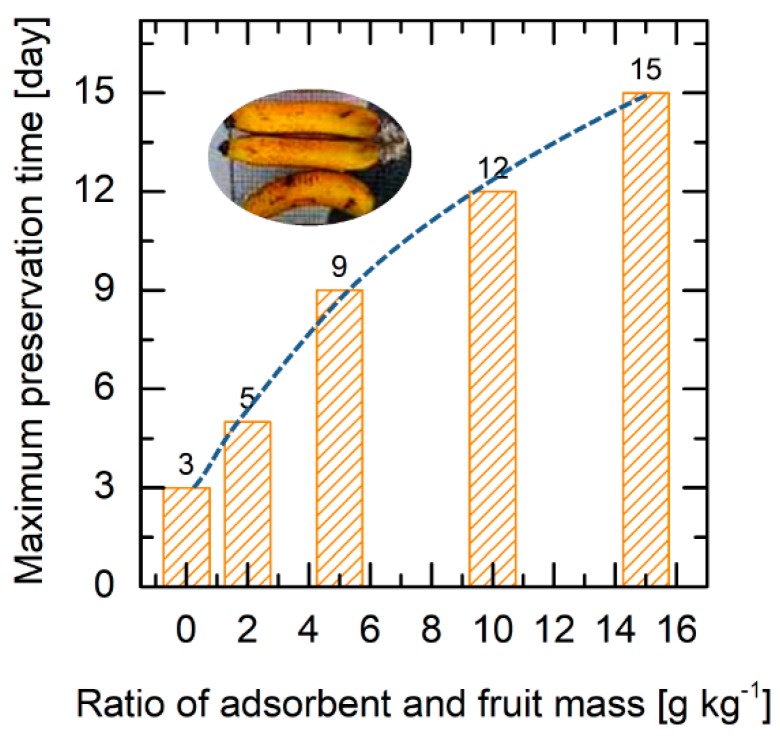
The effect of adsorbent amount on the maximum preservation time of fruit. Storage conditions: ratio of adsorbent and fruit varied, 25 °C temperature and 80% relative humidity.

To study the effect of the amount of adsorbent, the mass ratio of adsorbent and fruit was varied. Figure 5 displays the preservation time vs. mass ratio of adsorbent to fruit in the range of 0–16 g kg^−1^. The amount of adsorbent affects the shelf life, and a non-linear correlation is seen (a representation, see the dashed line in Figure 5). Without adsorbent, the storage time is only 3 d. The shelf life can be extended from 2 to 12 d depending on the mass of adsorbent added. By adding 15 g adsorbent for 1 kg fruit, shelf life enhancement up to 5-fold can be achieved. A higher mass of adsorbent provides a longer preservation time which is likely caused by a higher content of active sites for ethylene adsorption. The results show the promising application of cobalt oxide-impregnated nanoporous carbon for extending the shelf life of fruit.

## 3. Materials and Methods 

### 3.1. Nanoporous Carbon Preparation

Porous carbons were synthesized by pyrolysis, a natural source of mangosteen shells (Bina Agro Mandiri, Indonesia) [9,10], and a synthetic polymer of resorcinol-(*para*-tert-butyl phenol)-formaldehyde. Initially, the polymer was placed in a high temperature furnace (JSR, Gongju-City, Korea). In order to remove the oxygen/air content, the furnace was flushed sufficiently with nitrogen (inert gas, 99%, PT Samator, Indonesia). The system was then heated to 850 °C with ramp rate of 0.083 °C s^−1^ under the flow of nitrogen, and held at 850 °C for 3 h for the complete carbonization process. Subsequently, the furnace was cooled down to ambient temperature overnight under nitrogen purge. The resulting carbon materials from mangosteen shell biomass (natural polymer) and synthetic polymer were labeled as biomass-derived carbon and polymer-derived carbon, respectively. For comparison of carbon qualities, a commercial activated carbon based on coconut shell available from PT Home System Indonesia was also used. To prepare the synthetic polymer, polycondensation reaction was carried out as described elsewhere in [30]. 

### 3.2. Cobalt Oxide-Impregnated Nanoporous Carbon Preparation

Cobalt oxide was dispersed in porous carbon by the incipient wetness method followed by calcination process. Firstly, carbon was degassed at 150 °C for 2 h to clean the pores. Subsequently, salt solution of cobalt nitrate (99.8% purity) from Merck (Darmstadt, Germany) in isopropanol was added. After evaporation of solvent, the calcination process was then carried out by heating the mixture of carbon and cobalt nitrate to 200 °C under nitrogen purge in a quartz tubular reactor with the ramp rate of 0.033 °C s^−1^. The calcination follows Equation (2) [31]. According to the literature, the phase of cobalt oxide produced under these conditions is Co_3_O_4_ [32].

(2) $$3[\mathrm{Co}{\left({\mathrm{NO}}_{3}\right)}_{2}\;6\left(H_{2}O\right)]\overset{N_{2}flow}{\underset{heat}{\to }}{\mathrm{Co}}_{3}O_{4}+6{\mathrm{NO}}_{2}+O_{2}+18H_{2}O$$

The target of cobalt in carbon was set to 5, 10, 15, 20, and 30 wt.% (weight percentage) by proper addition of cobalt precursor (*m_precursor_*), determined by mass balance calculation as shown by Equation (3).

(3) $$m_{precursor}=\left(\frac{MW_{precursor}}{MW_{Co}}\right)\frac{Xm_{C}}{\left(1-X\right)}$$

where *MW* is the molecular weight, *X* is the cobalt content, and *m_c_* is the mass of carbon support. The resulting cobalt oxide-impregnated porous carbon was named by *X* wt.% Co/carbon. For instance, 10% Co/PDC refers to a 10% weight loading of cobalt on the polymer-derived carbon.

### 3.3. Characterization Methods

The pore structure of carbon was analyzed by nitrogen physisorption at −196 °C using liquid nitrogen as a coolant in NOVA 2000 (Quantachrome, Boynton Beach, FL, USA). Prior to the measurement, the sample was degassed up to 10^−3^ kPa at 125 °C for 8 h. The porous material was then filled with nitrogen through an increase of pressure from 0 to 1 (P/P_o_), hence obtaining the adsorption isotherm. Subsequently, the process was reversed to remove nitrogen in the pores to obtain the desorption branch. The pore size distribution was calculated from adsorption/desorption isotherms using the Quenched Solid Density Functional Theory model for slit/cylindrical-shaped pores [33] provided by the software NovaWin 11.03 (Quantachrome Instruments, Boynton Beach, FL, USA). The *T*-plot method was employed to determine the mesopore contents of surface area and pore volume as described elsewhere [34]. The scanning electron microscopy was taken to observe the morphology of the materials, using JSM-6510 LA (JEOL, Tokyo Japan) at 15 kV accelerating voltage equipped with an EDAX X-ray detector.

### 3.4. Ethylene Adsorption Measurement

**Figure 6:**
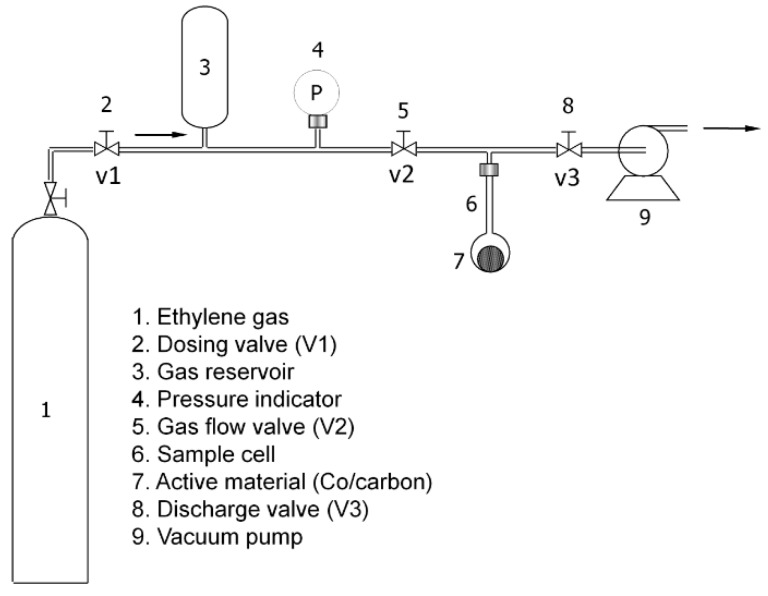
The schematic diagram of the adsorption rig employed for the ethylene adsorption test.

Ethylene (99.99%, PT Samator Indonesia) was used for the adsorption test. The uptake capacity was determined using a volumetric method in an ultra-high vacuum rig. The adsorption temperature was kept constant at 25 °C. The schematic diagram of the adsorption equipment is shown in Figure 6. The adsorption measurement apparatus was constructed by Swagelok VCR^®^ fittings (Singapore) and supplied by PT Putranata Adi Mandiri, Jakarta, Indonesia. About 1 g of cobalt oxide-impregnated carbon (7) was placed in the sample cell (6) and the system was firstly degassed at 150 °C overnight (valve V1 closed while V2 and V3 opened) until the static pressure in the system was at least 10^−3^ kPa as shown in the pressure indicator (4). The type of pressure indicator was a 910 DualTrans (MKS, Singapore). After ensuring the tightness of the system, the ethylene gas was gradually dosed from the ethylene bottle (1) to the adsorption rig. Isotherms of ethylene were recorded in the range of 0–101 kPa.

The amount of ethylene adsorbed (Δ*n*) was calculated using Equation (4).

(4) $$\Delta n=\frac{\left(P_{1}V_{1}-P_{2}V_{2}\right)}{RT}$$

where *P*_1_*V*_1_ and *P*_2_*V*_2_ represent the initial amount of ethylene dosed to the system and the amount of ethylene remaining in the gas phase in the equilibrium state, respectively. *R* is the gas constant and *T* is the temperature. The adsorbed ethylene was then converted to volume adsorbed at 0 °C and 1 atm as the standard temperature and pressure using the ideal gas law.

### 3.5. Preservation Test

The fruit preservation study was performed in a batch process. Cavendish bananas (*Musa acuminata*) purchased from Superindo Market (Sleman, Indonesia) as a fruit model was placed in a sample box and the cobalt oxide-impregnated nanoporous carbon (15%Co/PDC) was then placed in the system. The mass ratios of adsorbent and fruit were varied at 0, 2, 5, 10, and 15 g kg^−1^. The ambient temperature was at 25 °C and 80% relative humidity. Sample images of fruit were taken using a SELP1650 camera (Sonny, Japan). 

## 4. Conclusions

Porous carbons produced by pyrolysis of biomass of mangosteen shell and synthetic polymer showed different characteristics of pores. Biomass-derived carbon exhibited dominantly mesopores (90%) with specific surface area of 1080 m^2^ g^−1^, while polymer-derived carbon displayed a high content of micropores (60%) but a higher surface area (2390 m^2^ g^−1^). Cobalt substances could be successfully loaded in these porous carbons as proven by material characterizations. Good dispersion of cobalt oxide is achieved by conducting a two-step process of impregnation and calcination. The uptake capacity measurements exhibited that ethylene is more favorably adsorbed in cobalt oxide dispersed on carbon material possessing a high specific surface area. The volume of ethylene adsorbed increases with the increasing cobalt oxide loading on the carbon support. The highest adsorption capacity up to 350 cm^3^ g^−1^ at 101 kPa was obtained. The test showed that the adsorbent could extend the ripening process of Cavendish banana up to 12 days with respect to without adsorbent. The study showed a promising application of the adsorption method as an economical and effective method for ethylene removal. In addition, the system could also lead to the development of an adsorptive method for other horticulture products, such as vegetables.

## 5. Patents

Patent of cobalt oxide/polymer-derived carbon was registered (No. P00201603184) under the Ministry of Law and Human Rights of Republic Indonesia.

## Figures and Tables

**Table 1:** Pore parameters calculated from N_2_-adsorption desorption isotherm.

Porous Textural Parameter	*SSA*^a^, m^2^ g^−1^	%*SSA_Mic_* ^b^, %	*V_T_*^c^, cm^3^ g^−1^	%*V_mic_* ^d^, %	*d_v_*^e^, nm
Commercial activated carbon	1050	55	0.46	82	1.75
Biomass-derived carbon (BDC)	1080	32	1.53	10	5.66
Polymer-derived carbon (PDC)	2390	84	1.21	60	2.03
15% Co/PDC	1469	92	0.70	70	1.92
30% Co/PDC	928	87	0.49	63	2.10

^a^ Specific surface area, determined by multipoint Brunauer–Emmett–Teller (BET). ^b^ Micropore surface area fraction, determined by the *t*-plot method. ^c^ Total pore volume at 0.995 P/P_o_. ^d^ Mean pore diameter, calculated with $d_{v}=\frac{4V_{T}}{area}$. ^e^ Micropore volume fraction, determined by the *t*-plot method.

**Table 2:** Constant parameters of the Toth equilibrium model determined from ripening hormone adsorption data on various cobalt oxide-impregnated nanoporous carbons.

Parameter	Cobalt Oxide/Biomass-Derived Carbon					Cobalt oxide/Polymer-Derived Carbon		
	Blank BDC	5%Co/BDC	10%Co/BDC	20%Co/BDC	30%Co/BDC	Blank PDC	15%Co/PDC	30%Co/PDC
*C_µs_*, cm^3^ g^−1^	76.57	147.08	159.35	164.38	195.51	174.24	273.10	553.70
*b*, kPa^−1^	0.01	0.06	0.08	0.10	0.27	0.01	0.92	1.76
*t*, -	0.94	0.67	0.65	0.66	0.51	0.95	0.39	0.35

## Supplementary Materials

The following are available online at https://www.mdpi.com/1420-3049/24/8/1507/s1, Figure S1: Adsorption of ethylene using adsorbent of 5% Co/C as function of temperature, Figure S2: Repeatability test of adsorbent at equilibrium conditions of 101 kPa and 25 °C.
