# Supplementary material for: Ethylene Adsorption Using Cobalt Oxide-Loaded Polymer-Derived Nanoporous Carbon and Its Application to Extend Shelf Life of Fruit
Source: Molecules. 2019 Apr 17;24(8):1507. doi: 10.3390/molecules24081507 (PMC6514864; doi:10.3390/molecules24081507)
Supplement: Supplementary file 1 [file molecules-24-01507-s001.pdf]

Supplementary Material

Adsorptive removal of ethylene using cobalt oxide-impregnated nanoporous carbon and its application to extend shelf life of fruit

Imam Prasetyo [a,b]*, Nur Indah Fajar Mukti [c], Teguh Ariyanto [a,b]*

[a] *Department of Chemical Engineering, Universitas Gadjah Mada, 55281 Yogyakarta, Indonesia*

[b] *Advanced Material and Sustainable Mineral Processing Research Group, Universitas Gadjah Mada, 55281 Yogyakarta, Indonesia*

[c] *Department of Chemical Engineering, Islamic University of Indonesia, 55584Yogyakarta, Indonesia*

*Corresponding authors: imampras@ugm.ac.id; teguh.ariyanto@ugm.ac.id;

Data of effect of temperature on ethylene adsorption

Adsorption of ethylene in Co/carbon at different temperature (293-313 K) was carried out to observe interaction between cobalt oxide and ethylene. The results showed that ethylene removal is more favorable at higher temperature suggesting chemisorption interaction between ethylene and cobalt oxide. The enthalpy of adsorption was evaluated by Henry constant ($C\mu = kP$) at low adsorption pressure (<0.2 bar). The value of enthalpy of adsorption is 74.1 kJ/mol which in the range of chemisorption.

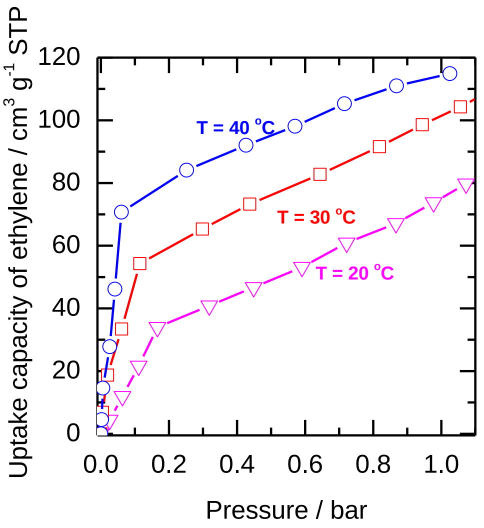

Figure S1. Adsorption of ethylene using adsorbent of 5% Co/C as function of temperature

Data of Adsorbent Repeatability

Adsorbent of 15%Co/PDC was tested to adsorb ethylene using a volumetric method as described in the Methodology. The target equilibrium pressure was 101 kPa at 25 °C. The uptake capacity was ca. 170 cm^3/g adsorbent. This value was set as initial uptake capacity ($C_{\mu 0}$) of Cycle 1. Ethylene in the adsorbent was then removed by degassing process at 250 °C under vacuum condition for one day. After that, the adsorption of ethylene was further

performed until reaching equilibrium at 101 kPa. The uptake capacity of this Cycle 2 was then recorded and rationalized using the initial uptake capacity. The experiment of Cycle 3 was identical to procedure of Cycle 2.

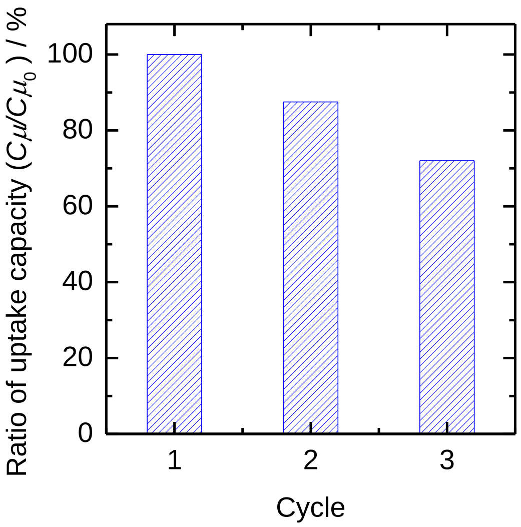

Figure S2. Repeatability test of adsorbent at equilibrium conditions of 101 kPa and 25 °C
